# No rapid audiovisual recalibration in adults on the autism spectrum

**DOI:** 10.1038/srep21756

**Published:** 2016-02-22

**Authors:** Marco Turi, Themelis Karaminis, Elizabeth Pellicano, David Burr

**Affiliations:** 1 Department of Translational Research On New Technologies in Medicine and Surgery, University of Pisa; 2 Centre for Research in Autism and Education (CRAE), UCL Institute of Education, University College London, UK; 3 Department of Neuroscience, Psychology, Pharmacology and Child Health, University of Florence, Italy; 4 School of Psychology, University of Western Australia, Perth, Australia

## Abstract

Autism spectrum disorders (ASD) are characterized by difficulties in social cognition, but are also associated with atypicalities in sensory and perceptual processing. Several groups have reported that autistic individuals show reduced integration of socially relevant audiovisual signals, which may contribute to the higher-order social and cognitive difficulties observed in autism. Here we use a newly devised technique to study instantaneous adaptation to audiovisual asynchrony in autism. Autistic and typical participants were presented with sequences of brief visual and auditory stimuli, varying in asynchrony over a wide range, from 512 ms auditory-lead to 512 ms auditory-lag, and judged whether they seemed to be synchronous. Typical adults showed strong adaptation effects, with trials proceeded by an auditory-lead needing more auditory-lead to seem simultaneous, and *vice versa*. However, autistic observers showed little or no adaptation, although their simultaneity curves were as narrow as the typical adults. This result supports recent Bayesian models that predict reduced adaptation effects in autism. As rapid audiovisual recalibration may be fundamental for the optimisation of speech comprehension, recalibration problems could render language processing more difficult in autistic individuals, hindering social communication.

Autism is a heritable, lifelong neurodevelopmental condition with striking effects on social communication. The condition is also associated with a range of non-social features, including both *hyper*sensitivity and *hypo*sensitivity to perceptual stimuli, and sensory seeking behaviours such as attraction to light, intense looking at objects and fascination with brightly coloured objects. These sensory atypicalities, which now form part of the diagnostic criteria for autism^1^, can have debilitating effects on the lives of autistic people and their families^2^,^3^.

To create a unified percept of the world, the brain has to combine multiple sources of sensory information into one coherent multisensory percept. This is particularly important for speech, which in noisy environments is easier to understand when the speaker’s lip movements can be observed^4^. Combining visual and auditory signals is not a trivial task, given both the difference in the transmission speed of light and sound and variable neural delays^5^. To keep the auditory and visual information temporally aligned, the brain must continually recalibrate. There is a good deal of evidence for active recalibration: after repeated exposure to an asynchronous audiovisual asynchrony, synchronous signals appear asynchronous, in the other direction^6^,^7^,^8^,^9^. Although most adaptation studies use prolonged exposure to asynchronous audiovisual signals, Van der Burg *et al.*^10^ have introduced a new technique showing that rapid adaptation to asynchronous audiovisual events, occurs more rapidly than was previously thought, even with audiovisual speech^11^. They presented participants with a stream of audiovisual events with variable asynchrony and asked them to judge if they appear synchronous. Perceived synchrony depended on the temporal order of the preceding stimulus, demonstrating instantaneous adaptation after a single presentation (see also^12^).

Difficulties in multisensory processing, particularly early in development, might lead to problems in social and adaptive behaviour and interpersonal interactions that occur in autism. Some evidence for such difficulties comes from studies on multisensory integration of speech and emotions, perceived from the face and the voice^13^,^14^,^15^,^16^,^17^,^18^,^19^. These studies suggest that autistic people have specific problems with audiovisual integration of social and emotional stimuli, which could contribute to their social difficulties. In general, these studies show that autistic people have poorer multisensory temporal acuity, a finding that typically manifests as a broadening of their multisensory temporal binding window^13^,^20^,^21^,^22^,^23^. However, it appears that multisensory binding is affected primarily for linguistic stimuli, with relative sparing of low-level integration^16^; and significant group differences in audiovisual ERPs occur in the time window when spoken words begin to be processed for meaning^17^. Only one study has shown differences with simple, non-linguistic stimuli: autistic participants use audiovisual synchrony to a lesser extent to aid in “pop-out” visual search tasks^24^. Given the importance of multisensory interaction for communication, it is possible that atypical multisensory integration could underlie some of the perceptual and cognitive differences associated with autism.

We have recently proposed a Bayesian account of autism^25^ to explain such differences, suggesting that it is not sensory processing itself that is disrupted in autism, but the interpretation of the sensory input. The Bayesian class of theories – including predictive coding and other generative models^26^,^27^,^28^ assume that perception is an optimized combination of external sensory data (the *likelihood*) and an internal model (the *prior*). We suggested that this process may be atypical in autism, in that the internal *priors* are under-weighted, less utilized than in typical individuals. Our theory has been followed by several others along similar lines^29^,^30^,^31^,^32^,^33^.

The suggestion of under-utilization of priors leads to several specific predictions. One strong prediction is that autistic individuals should show reduced adaptation aftereffects; and much data shows reduced adaptation in autism for high-level features, both social (e.g., faces^34^) and non-social (e.g., numerosity^35^). Adaptation helps to improve neuronal efficiency by dynamically tuning its responses to match the distribution of stimuli to make maximal use out of the limited working range of the system^36^,^37^,^38^. Failure to continuously adapt to the current environment should lead to inefficiencies, including the transmission of redundant information, which could have profound effects for how an individual might perceive and interpret incoming sensory information.

In this study we examine whether autism is also associated with reduced adaptation for multisensory stimuli. Specifically, we test whether adaptation to simple, non-linguistic audiovisual asynchrony is reduced in autistic people, using the robust and rapid technique of Van der Burg *et al.*^10^. We find robust recalibration in typical adults, very similar to published results^10^, but our autistic participants showed no trial-wise recalibration.

## Results

**Figure 1:**
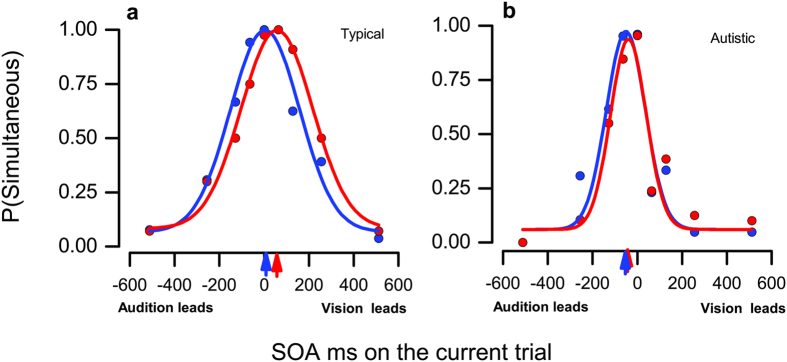
Effect of previous trial on simultaneity judgments. Proportion of trials judged to be simultaneous as a function of Stimulus Onset Asynchrony (SOA) for two representative participants, one typical adult (**a**), one autistic adult (**b**). Negative SOAs indicate the tone preceded the luminance onset, positive that the tone followed the luminance onset. The data were divided into those where audition leads in the previous trial (blue symbols) and those where it lags (red symbols), ignoring trials preceded by simultaneous audiovisual stimuli. Colour-coded curves are best-fitting Gaussian distributions, free to vary in mean (giving and estimate of PSS) and standard deviation (estimating simultaneity bandwidth).

We measured dynamic adaptation to audiovisual stimuli in 16 autistic adults and 16 typical adults, asking them to judge whether a briefly flashed white ring appeared perceptually simultaneous with an auditory tone. The ring and tone were presented with variable asynchrony between ±512 ms (see methods). For each participant, we separated the data into trials where the sound led in the *previous* trial (blue symbols, Fig. 1), and those where it lagged (red symbols), ignoring trials preceded by zero delay. Proportion of trials judged simultaneous was plotted as a function of stimulus onset asynchrony (SOA), and fit by a gaussian free to vary in mean and standard deviation. Figure 1 shows the procedure for representative typical (a) and autistic (b) participants. The typical adult shows the adaptation effect, with the simultaneity judgments of the audio-lead trials (blue) being fit by a gaussian centred about 53 ms more negative than the auditory-lag trials (red). That is, if preceded by an audio-lead stimulus, the audio in current stimulus had lead vision more than on the other trials to be perceived as simultaneous, implying a negative adaptation aftereffect. However, for the autistic adult, the curves were superimposed, with no measurable displacement.

**Figure 2:**
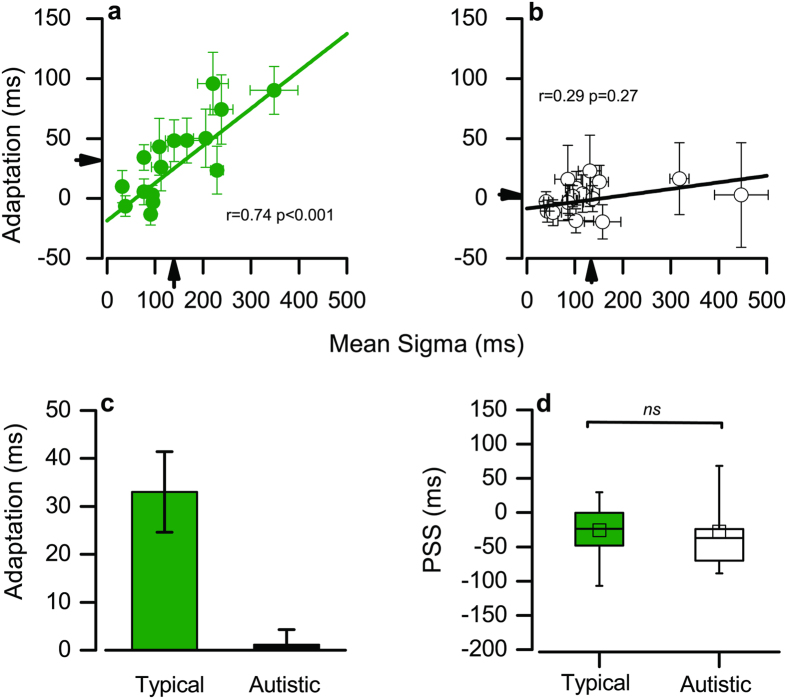
Adaptation effect, point of perceived simultaneity and simultaneity bandwidths. (**a**,**b**) The adaptation effect (the difference in PSS for trials preceded by audio-lead and those by audio-lag) as a function of the simultaneity bandwidth (standard deviation of Gaussian fitted to all data), for typical (**a**) autistic (**b**) adults. Data were bootstrapped to estimate standard errors, indicated by the bars. (**c**) Mean adaptation effect for the two groups. (**d**) PSSs for each group, given by the mean of the Gaussian functions fitted to all data of each participant. The box refers to the 25 and 75 percentiles, the whiskers to 5 and 95 percentiles.

Figure 2 shows the adaptation effect (difference between the means of the fitted gaussians for auditory-lead and auditory-lag) for each individual, plotted against their simultaneity bandwidths (standard deviation of gaussian fitted to all trials). While both groups have a similar range of simultaneity bandwidths (arrows indicate means; t_(30)_ = 0.21 p = 0.83, two-tailed), only the typical adults show strong adaptation effects (see arrows on ordinate, and bar graphs of Fig. 2c; t_(30)_ = 3.54, p = 0.001, two-tailed). When we remove the two autistic participants with especially high bandwidths (see Fig. 2b), there are still no significant group differences in participants’ bandwidths, t^(28)^ = 1.71 p = 0.10, two-tailed).

As Van der Burg *et al.*^10^ observed, for the typical adults, there is a robust correlation between strength of adaptation and simultaneity bandwidth, with participants with broader simultaneity bandwidth showing greater adaptation effects (r = 0.74, p < 0.001). However, for the autistic adults, there was no measurable relationship (r = 0.29, p = 0.27).

Figure 2d shows the PPSs (points of subjective simultaneity) for typical and autistic participants, given by the means of gaussians fitted to all data from each participant. The mean for both groups is about −25 ms, implying that the auditory stimulus had to lead by 25 ms for the two to appear simultaneous. There is considerable spread in PSS within both groups, covering a similar range (see box and whisker plot), but no significant difference between the groups (t_(30)_ = 0.1, p = 0.92, two-tailed).

**Figure 3:**
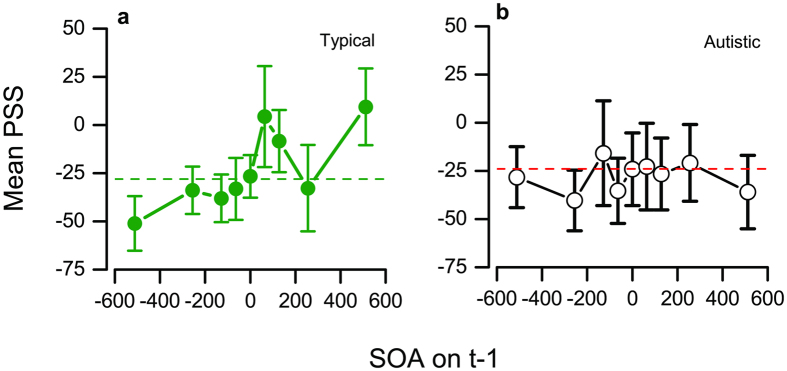
Effect of SOA of previous trial on point of subjective simultaneity. Mean PSS (calculated separately for each participant, than averaged) as a function of SOA of the previous trial, for the typical group (**a**) and autistic group (**b**).

Figure 3 examines how PSS varies as function of the SOA on trial t−1 (again calculated separately for each participant, then averaged). For the typical group, there was a clear dependency on SOA, F(8,120) = 2.38 p = 0.02, but none for the autistic group, F(8,120) = 0.60, p = 0.77. It is evident that the dependency on previous trials occurs principally when the previous trial had a very large lead or lag, at ± 512 ms.

**Figure 4:**
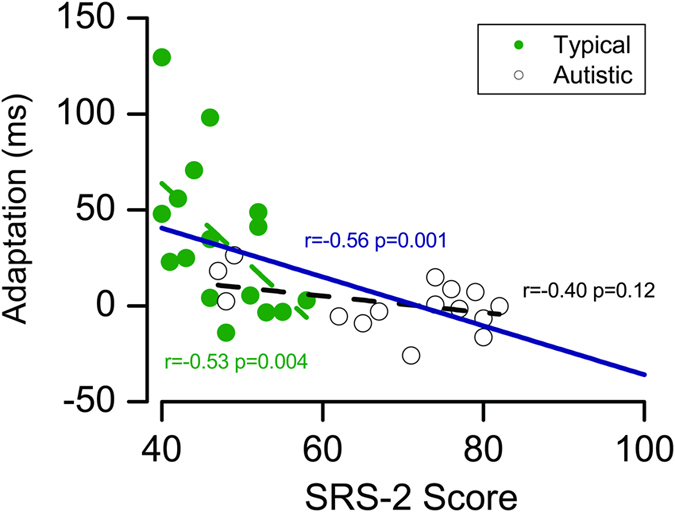
Relationship between adaptation and Social Responsiveness Scale. Adaptation effect (the difference in PSS for trials preceded by audio-lead and those by audio-lag) as a function of Social Responsiveness Scale (SRS-2) score for all individuals (typical: green; autistic: black). The colour-coded broken lines show the correlations within each group, the continuous navy line the correlation between all participants. Full (blue dashed line) and partial correlation between recalibration effect and autistic traits measured by SRS-2 in the typical (green dashed line) and autistic (black dashed line) group.

Figure 4 plots the magnitude of the adaptation effect against SRS-2 scores. Considering all participants, the correlation was strong and significant (r = −0.56, p = 0.001). Interestingly, the correlation was also significant within the typical group (r = −0.53, p = 0.04), showing that reduced adaptation co-varies with autistic traits, even for individuals not diagnosed with autism. Within the autistic group, there was a non-significant trend (r = −0.40, p = 0.12). Given that the adaptation for all the autistic participants was close to zero, the lack of significance most likely reflects a floor effect.

## Discussion

The results clearly show that autistic adults do not rapidly adapt to audiovisual synchrony, although matched typical adults showed robust adaptation. Furthermore, while the strength of adaptation in typical adults correlated strongly with the bandwidth of the perceived audiovisual synchrony window, there was no such correlation in the autistic group.

These results fit well with recent Bayesian models of autism^25^,^29^,^32^,^33^ that predict that individuals with autism should give less weighting to *prior* or *predictive* information and have reduced adaptation effects. While the mechanisms of adaptation may not be fully understood, adaptation is one of the clearest examples of transient neural plasticity, where the output of perceptual processes depend not only on the current stimuli, but on the immediate history. Many models link adaptation effects to Bayesian prediction^39^,^40^, suggesting that *priors* may serve as standards for self-calibration, which is the function of adaptation. Atypicalities in the prior in autistic individuals – either in its construction or use as a calibration standard – should impact on the magnitude of adaptation.

Previous research has demonstrated reduced adaptation in autism to faces^34^ and to number^35^, but not to more basic properties such as motion and shape aftereffects^41^,^42^. Similarly, in audition, autistic participants show reduced “simple loudness adaptation”, thought to be mediated by the central auditory system, but normal “induced loudness adaptation”, thought to be mediated by the peripheral auditory system^43^. It seems that reduced adaptation for high-level attributes is a general property of autistic perception. Both face and number perception are complex but important perceptual processes, where adaptation is thought to serve an important recalibration function^44^,^45^. Audiovisual synchrony must also involve higher-level processes, after the separate processing of auditory and visual signals.

There is considerable evidence that integration of audiovisual linguistic stimuli is atypical in autism^13^,^14^,^15^,^16^,^17^,^18^,^19^ but the underlying neural mechanisms for the atypicalities are not understood. Observing lip movement helps speech understanding, but only if the two are synchronized^4^. Detecting audiovisual synchrony is therefore fundamental for integration of audiovisual linguistic stimuli. This is a complex task for the nervous system. As both signals are subject to variable delays, it is not sufficiently simply to judge which stimulus arrived first at a given neural station, but constant recalibration is necessary to deal with the variability in timing. There is much evidence for audiovisual recalibration in typical adults, both after lengthy presentations of asynchronous stimuli^6^,^7^,^8^,^9^, and even after a single, brief exposure^10^,^11^,^12^,^46^.

Here we show that autistic adults do not show the rapid adaptation effects that we reliably reproduce in typical adults. There was no measurable adaptation in our group of autistic participants, despite using the identical procedure to that used for the typical participants, and in previous studies^10^. Furthermore, there was strong correlation between adaptation strength and scores on the social responsiveness scale (SRS-2). This correlation was driven mainly by the typical adults, suggesting that an association between lack of rapid adaptation and autistic traits exists, even for individuals below the diagnosis threshold. The lack of correlation within the autism group probably results from saturation of the effect: adaptation was near zero for the entire group.

Importantly, besides the lack of rapid adaptation, there were no obvious differences between the two groups in the perceived synchrony tasks other than the absence of adaptation. The mean point of subjective synchrony was very similar for the two groups, both about −25 ms (audio-lead), comparable spread of PSSs between individuals. The widths of the simultaneity window were also similar for the two groups, both the mean and the variability: the only difference was that in the group of autistic adults, the widths did not correlate with adaptation strength. All of this suggests that, despite differences in rapid adaptation, autistic people face no gross problems in perceiving synchrony of non-linguistic audiovisual signals. Our results are in line with studies reporting no significant difference in the width of simultaneity window in autistic people using non-linguistic stimuli^13^,^18^,^23^. Perhaps they have other mechanisms involved in the conscious perception of audiovisual synchrony; or perhaps other calibration mechanisms are involved, operating over a longer timescale. It would be interesting to measure perceived synchrony under variable conditions, such as distance and luminance, both of which should affect neural synchrony. Interestingly, the only other evidence of autism-related differences in audiovisual integration with non-linguistic stimuli is the reduced audiovisual “pop-out” in visual search^24^, where temporal coincidence in sounds and visual targets makes them more detectable. Perhaps this task relies on rapid calibration mechanisms.

Although the lack of rapid recalibration seems to have no gross consequences to audiovisual synchrony judgements of simple, non-linguistic stimuli in autistic adults, it may have more subtle consequences, which could be important, for example, for speech perception. There are indications that this may be the case. While most studies have found no problems in audiovisual integration of simple stimuli^13^,^18^, there is both psychophysical and electrophysiological evidence that autism is associated with reduced audiovisual integration in speech perception^16^,^17^. It may be that one aspect contributing to the reduced audiovisual speech integration and lip-reading abilities^19^ is the failure to keep audiovisual synchrony calibrated. As the integration of lip movements and speech is greatest when the inputs are synchronized, rapidly aligning temporally offset auditory and visual streams after brief asynchrony would be extremely beneficial. For example, rapid recalibration would ensure that an audiovisual speech stream received from a source at any distance could be rapidly realigned to maximize speech comprehension.

In the case of reduced adaptation in autism, as shown here, the efficiency that would be gained from processing multiple sensory signals as a single percept driven by recalibration would be lost, resulting in less efficient sensory processing overall. Further investigation is needed to better understand the neural substrates for this mechanism and their relationship with audiovisual integration in speech perception.

## Methods

### Participants

We tested 16 autistic adults and 16 typical adults (with no current or past medical or psychiatric diagnosis), matched for age (t_(30)_ = 1.38 p = 0.17, two-tailed), gender and intellectual functioning (t_(30)_ = −0.44 p = 0.65, two-tailed) (see Table 1). All autistic adults had received a clinical diagnosis of autism or Asperger’s disorder according to Diagnostic and Statistical Manual of Mental Disorders, Fourth Edition (DSM-IV) criteria^1^ from independent clinician, and also met criteria for an autism spectrum disorder (ASD) on the Autism Diagnostic Observation Schedule – 2^nd^ edition (ADOS-2)^47^. No participant had a medical or developmental disorder (other than ASD), nor was on medication, and all reported normal visual acuity and hearing. All participants completed the Social Responsiveness Scale – 2^nd^ Edition (SRS-2)^48^, a quantitative measure of autistic traits. Participants were seen individually in a quiet room at the university. All procedures were approved by the regional ethics committee at the *Azienda Ospedaliero-Universitaria Meyer*. The participants gave their informed consent prior to their participation in accordance with the institutional approved guidelines.

### Stimuli and Procedure

Stimuli were generated with the Psychophysics Toolbox^49^ and presented at a viewing distance of 57 cm on a 23” LCD Acer monitor (resolution = 1920 × 1080 pixels; refresh rate = 60 Hz; mean luminance = 60 cd/m^2^), run by Macintosh laptop. The visual stimulus was a white ring (radius 2.6°; width 0.4°), presented around a white fixation cross on a black background (60 cdm) for a duration of 50 ms. The auditory stimulus was a 500 Hz tone of 50 ms duration, presented via headphones (Sennheiser HD201). Each trial started with the white fixation cross on a black background for 1 s, followed by an audiovisual stimulus of variable delay, randomly 0, ±64, ±128, ±256, ±512 ms, where negative means the auditory stimulus was presented first. Participants did not judge temporal order; rather, they were instructed to judge (by corresponding key press) whether the sound and visual display appeared to be synchronous or not. Participants performed 40 trials for each temporal condition in four sessions, giving a total of 400 trials. Fixation was monitored by the experimenters (two were present for all testing sessions).

## Additional Information

**How to cite this article**: Turi, M. *et al.* No rapid audiovisual recalibration in adults on the autism spectrum. *Sci. Rep.* **6**, 21756; doi: 10.1038/srep21756 (2016).

## Figures and Tables

**Table 1:** Descriptive statistics for developmental variables for autistic and typical adults.

	Autistic adults	Neurotypical adults
N	16	16
Gender (male: female)	12: 4	13: 3
Age (years)		
Mean (SD)	29 .2 (5.2)	27.1 (2.83)
Range	17–34	18–31
Full-Scale IQ^a^		
Mean (SD)	112.0 (10.32)	112.7 (11.44)
Range	89–129	94–139
ADOS-2^b^		
Mean (SD)	8.56 (2.03)	—
Range	7–13	*—*
SRS-2^c^		
Mean (SD)	69.37 (12.07)	47.31 (5.61)
Range	47–74	40–58

^a^Full-Scale IQ was measured using the Wechsler Abbreviated Scales of Intelligence^50^.

^b^ADOS-2: Autism Diagnostic Observation Schedule – 2nd Edition^47^; Higher scores reflect increased autistic symptomatology.

^c^Social Responsiveness Scale, Second Edition (SRS-2)^48^.
